# Pregnant during the COVID-19 pandemic: an exploration of patients’ lived experiences

**DOI:** 10.1186/s12884-021-04337-9

**Published:** 2021-12-31

**Authors:** Sabrina Kolker, Anne Biringer, Jessica Bytautas, Haley Blumenfeld, Sahana Kukan, June C. Carroll

**Affiliations:** 1 Ray D. Wolfe Department of Family Medicine, Sinai Health, 60 Murray Street, Box 25, Toronto, ON M5T 3L9 Canada; 2 Department of Family and Community Medicine, University of Toronto, Toronto, ON Canada; 3 Dalla Lana School of Public Health, University of Toronto, Toronto, ON Canada

**Keywords:** Pregnancy, Mental health, COVID-19, Psychosocial behavior, Support

## Abstract

**Background:**

Infectious outbreaks are known to cause fear and panic. Exploration of pregnant individuals’ psychosocial condition using a qualitative lens during an infectious outbreak is limited. In this study we explore pregnant individuals’ lived experiences as well as their psychological and behavioural responses during COVID-19 with the goal of providing useful strategies from the patient’s perspective to enable health care providers to help pregnant patients navigate this and future pandemics.

**Methods:**

Pregnant individuals between 20-weeks gestation and 3 months postpartum who received maternity care from an urban academic interprofessional teaching unit in Toronto, Canada were invited to participate. Semi-structured 60 min interviews were audio-recorded, transcribed and analyzed using descriptive thematic analysis. Interview questions probed psychological responses to the pandemic, behavioural and lifestyle changes, strategies to mitigate distress while pregnant during COVID-19 and advice for other patients and the healthcare team.

**Results:**

There were 12 participants, mean age 35 years (range 30–43 years), all 1 to 6 months postpartum. Six main themes emerged: 1) Childbearing-related challenges to everyday life; 2) Increased worry, uncertainty and fear; 3) Pervasive sense of loss; 4) Challenges accessing care; 5) Strategies for coping with pandemic stress; 6) Reflections and advice to other pregnant people and health care professionals. Pregnant individuals described lack of social support due to COVID-19 pandemic restrictions and a profound sense of loss of what they thought their pregnancy and postpartum period should have been. Advice to healthcare providers included providing mental health support, clear and up to date communication as well as more postpartum and breastfeeding support.

**Conclusions:**

These participants described experiencing psychosocial distress during their pregnancies and postpartum. In a stressful situation such as a global pandemic, health care providers need to play a pivotal role to ensure pregnant individuals feel supported and receive consistent care throughout the pregnancy and postpartum period. The health care provider should ensure that mental health concerns are addressed and provide postpartum and breastfeeding support. Without addressing this need for support, parental mental health, relationships, parent-infant bonding, and infant development may be negatively impacted.

## Background

When the Coronavirus disease 2019 (COVID-19) was declared a global pandemic on March 11, 2020 by the World Health Organization (WHO) [1], exploration of pregnant patients’ psychological condition during an infectious outbreak was limited. What was known from the SARS epidemic was that pregnant individuals experienced anticipatory worry and heightened anxiety during an outbreak, feared antenatal visits and some cancelled or postponed tests [2]. As the widespread transmission of COVID-19 has increased, so has our understanding of the damaging effects of the pandemic on perinatal mental health. Studies have demonstrated pregnant and postpartum women are at increased risk for developing heighted anxiety, depression, post-traumatic stress disorder and suicidality due to the significant psychological toll of COVID-19 [3–6]. Although the public health mandated strategies (e.g., physical distancing) for limiting spread of the disease have had important consequences for all people, there may be wider implications for pregnant and postpartum individuals. Important contributors to favorable pregnancy outcomes including healthy nutrition, [7] regular exercise, [8] adequate sleep, [9] and regular prenatal visits [10], may be less accessible during the COVID-19 pandemic with consequences for both physical and mental health.

Concerns are being raised about the pandemic’s potential long term negative effects on both women’s and infants’ mental health [11]. Previous studies have shown that maternal stress and anxiety during the antenatal period are associated with poor neonatal outcomes, including preterm delivery, low birth weight, and complications such as preeclampsia [12–16]. The negative consequences of maternal anxiety may extend well past the neonatal period as several studies have demonstrated deleterious effects on childhood cognitive and emotional development [17–19]. Perinatal depression has also been associated with adverse child outcomes [20–26].

There is limited research using a qualitative lens to further understand pregnant individuals’ lived experience during the COVID-19 pandemic [27, 28]. The goal of this study was to explore pregnant and postpartum individuals’ lived experience during the COVID-19 pandemic to better understand their psychological and emotional responses and behaviors, with a focus on specific strategies to ameliorate distress.

## Methods

We used a qualitative descriptive approach to understand the lived experiences of pregnancy during the COVID-19 pandemic. Qualitative descriptive studies draw on the tenets of naturalistic inquiry to present low-inference interpretations of participants’ experiences in everyday language [29]. Qualitative description is particularly well-suited to obtaining straight or minimally-theorized answered to questions of relevance to practitioners and policy makers [30]. We chose interviews to enable individuals to privately describe their experiences.

### Participants and setting

All patients receiving maternity care from the Mount Sinai Academic Family Health Team (MSAFHT), who were between 20 weeks pregnant and 3 months postpartum at the time of the study (*n* = 200), were invited via email to complete a questionnaire between June 2020–September 2020. The MSAFHT is an academic family medicine teaching unit in Toronto, Canada where pregnant individuals receive prenatal, intrapartum and postpartum care by family physicians and other members of the interprofessional team. Email invitations were sent from an MSAFHT administrator to avoid the possibility of undue bias or coercion, as some patients may have been under the active care of three members of the study team (SKo, AB, HB). Upon completion of the questionnaire, participants were asked to indicate their willingness to participate in an interview by providing their email address. All participants who provided their email addresses were contacted by a team member with no prior relationship (JB) to arrange an interview.

### Data collection

An open-ended, semi-structured interview guide was developed by the interprofessional research team (family physicians and a qualitative health researcher) based on literature from previous pandemics and from our experience caring for pregnant individuals during the early stages of the COVID-19 pandemic. (Interview guide available upon request). Questions were created to explore participants’ experiences of pregnancy and the early postpartum period during the pandemic, perceived risks associated with pregnancy and COVID-19, behavioural responses, and strategies to manage worry and anxiety. The guide was modified following a pilot interview. The pilot interview was conducted with one participant who met our inclusion criteria of being pregnant or postpartum during the pandemic. The pilot interview was recorded, but not transcribed, for the purposes of refining the interview guide. Team members reviewed the pilot audio file and met to discuss modifications to the interview guide before proceeding with formal data collection. Pilot data were not included in the data analysis. The team met at two points during data collection to discuss preliminary findings and further refinements to the interview guide, after the completion of the third and seventh interviews.​

Interviews were conducted by phone by a team member with expertise in qualitative health research (JB). Interviews averaged 1 h and were conducted between July and October 2020.

### Data analysis

Interviews were audio-recorded and transcribed verbatim. Transcripts were stripped of identifying information and stored and managed using NVivo qualitative data analysis software (Version 12, QSR International). Transcripts were not returned to participants for comments and/or correction. We opted not to re-engage participants given the highly sensitive nature of the topic and the likelihood that participants may legitimately change their perspectives on the topic as time went by [31]. Further, we did not wish to presume that participants would have the ability or interest to comment productively on the scientific discourse [32].

We employed a qualitative descriptive approach to support rich description and low inference interpretation [29, 30]. Data were analyzed using techniques of constant comparison to search for thematic patterns and relations [33]. We used a mixed strategy for coding, allowing codes to emerge empirically from the data, but for some questions (particularly those about coping strategies and advice) we used predetermined codes from our interview guide. Coding was led by two researchers (SKo, JB), with all members of the study team meeting periodically to review sub-sets of transcripts to refine the coding framework and resolve discrepancies or disagreements in an iterative fashion through discussion.

### Ethical considerations

We conducted and present our study in accordance with the consolidated criteria for reporting qualitative research (COREQ) [34]. Ethics approval was obtained from the Mount Sinai Hospital Research Ethics Board (20–0123-E).

## Results

Nineteen patients indicated a willingness to participate and 12 completed an interview. All participants were between 3 weeks to 12 months postpartum by the time of the interview. Table 1 shows participant characteristics.

**Table 1:** Participant Demographics

Characteristic	Qualitative Participants (*n* = 12^a^)
Age (mean, range)	35 years (30–43)
Pregnancy Status	
• Post-partum	12 (100%)
Marital Status	
• Married/Common-Law/Living with Partner	11 (91.7%)
• Never Married	1 (8.3%)
Other Children	
• No	8 (66.7%)
• Yes	4 (33.3%)
Household Income	
• >$100,000	8 (66.7%)
• $50,000–$100,000	2 (16.7%)
• <$50,000	1 (8.3%)
• Prefer not to say	1 (8.3%)
Education	
• Graduate degree or professional training	7 (58.3%)
• Completed University or College	5 (41.7%)

^a^19 participants initially indicated willingness to participate, 12 participants completed the interviews

Six main themes emerged from our analysis of participants’ descriptions of their experience being pregnant and postpartum during COVID-19: 1) Childbearing-related challenges to everyday life; 2) Increased worry, uncertainty and fear; 3) Pervasive sense of loss; 4) Challenges accessing care; 5) Strategies for coping with pandemic stress; 6) Reflections and advice to other pregnant people and health care practitioners.

1. Childbearing-related challenges to everyday life

Participants described challenges to employment, finances, access to food and transportation. While many of these challenges may be common pandemic experiences, some were unique to pregnancy, and deeply affected the participants.

### Employment

Several of our participants worked in a health care setting and were working while pregnant during the pandemic. One participant described feelings of guilt for working in an at-risk environment, “Emotionally, I was drained and I was devastated because I needed to work, but I also needed to feel like I am not exposing my unborn baby, myself, and the little one at home.” [P2] Another participant described her feelings of fear while working in the hospital during the early stages of the pandemic, “I remember being quite pregnant, like around 33, 34 weeks pregnant, and we’re in the hospital and no one really knowing kind of how dangerous it was or what to do.” [P5] One participant contracted COVID-19 while working and another was potentially exposed while working. The participant who tested positive while pregnant noted even though her symptoms were mild, the perceived stigma and fear of returning to work “took a toll on me more mentally than physically.” [P2]

Even those not working in health care felt pressure to stop working as one participant explained, “And I started to get a lot of I guess pressure from not necessarily family, but friends of my husband and other people asking like why am I still working when COVID is everywhere.” [P10]

While the majority of participants had savings to rely on, some were deeply impacted financially due to employment cuts. As this participant describes, the financial strain took its toll on her relationship, which in turn could affect the baby: “with me not working and [husband] on part time hours, we definitely got hit really hard financially…when you have this kind of financial stress, everything is more stressful. It hurts your relationship. It makes the house more tense then that’s not good for the baby.” [P9]

At one point, another participant had to rely on a food bank and expressed concerns about nutrition for her and baby, “that was a hard one for me, because everything was tin – they don’t give you always veggies and stuff.” [P8]

### Access to food

Some participants created new routines to access food and delegated their partner as the sole person to go out to buy groceries, because of their perceived risk being pregnant or exposing their newborn baby. As one participant explained, “At the beginning it felt kind of scary…I didn’t want to take the baby to the grocery at all at first, so at the beginning my husband was doing all the grocery shopping.” [P1]

### Transportation

Traveling on public transit also caused increased stress due to the perceived risk of exposure while pregnant. One participant shared, “I felt really stressed out... I was having panic attacks on the subway.” [P12] Another postpartum participant decided to avoid taking the bus all together out of fear for her exposure, “You can’t take a bus because the busses are still packed. There’s no distancing on that. I have to take the baby, I’d rather not.” [P8]

2) Increased Worry, Uncertainty and Fear

Participants described a range of emotional responses to COVID-19. Most commonly these included worry, uncertainty and fear.

### Worry

Participants described worrying during the pandemic’s early stages as they tried to decrease potential exposure to the virus: “my partner…was still working full time and he didn’t have the option of taking the time off…we were concerned that if he caught it, he would bring it back to me and then subsequently me and the baby.” [P9] They also worried about what the birthing and visitor processes would be like and this caused increased anxiety, “I was very stressed about that in my pregnancy, very, and very upset at the thought of being alone.” [P4]

For some of the participants, medical testing was delayed and/or cancelled leading to anxiety waiting for results or worry about the baby. As one participant explained, “My genetics testing took longer long because there wasn’t any open.” [P3] Another participant described her worry during the postnatal period when her baby missed his hearing test, wondering if he would be OK, “all of these things were cancelled…is my baby going to be OK?” [P8]

### Uncertainty

The feeling of uncertainty was commonly expressed due in large part to the lack of knowledge about the effects of the virus on their health or their baby’s health. One participant reported, “initially when the pandemic started, it was obviously pretty scary, because I didn’t know much about the effects that COVID could have on pregnant women, on their babies.” [P2] Another participant also voiced her concerns about the potential risks to baby, “I remember, with the COVID stuff, feeling stressed in March, thinking could this be risky to the baby, or if I got sick would I go into pre-term labour or, you know, that kind of stuff.” [P5].

### Fear

Participants expressed fear about COVID-19’s contagious nature and potential harmful effects. One participant described fear causing panic when thinking about how quickly COVID-19 was spreading, “I expressed the fear so many times, you know, to see how far it has gone because one, I’m pregnant and I have a little kid at home, and I just hoped that it does not get worse…So it was scary, I’ll tell you that. It was not the best.” [P3]

3) Persistent and Pervasive Sense of loss

Participants reported a profound sense of loss due to COVID-19. Their experience of pregnancy and postpartum was not aligned with their hopes and dreams. One participant described, “It’s a loss that you cannot really talk to people that you feel you lost because you lost enjoying the pregnancy. You, in a nutshell, lose yourself. You know, in this whole scare, you know, trying to sanitize and do everything, you forget and you do not really experience your pregnancy yourself.” [P3]

They lost both tangible and emotional family support and expressed challenges to bonding with their baby.

### Expectations vs reality

Participants described that they had looked forward to spending time with others after the baby was born. One participant explained, “it seems like everything that you plan and everything that they tell you to do … You know, joining Mom and baby classes, and ask for help, and have people come over and all those things, it was just like OK, how do you deal when you suddenly can’t do any of those things?” [P1]

Many described a feeling of loss at not being able to celebrate their babies. One woman described feeling robbed as the celebrations for the baby both before and after birth did not occur as she had hoped, “yeah, definitely felt robbed of that. My mom had planned on having a party for everybody, the baby, which of course couldn’t happen and, yeah, those kind of things were kind of disappointing.” [P4]

### Bonding with baby

Participants expressed challenges bonding with the baby due to fears and anxieties generated from the pandemic as well as sadness that baby bonding with family would be delayed. “We had a name for [older son], but this baby, we didn’t have a name until like two weeks after it was born and we didn’t even engage ourselves with baby names and talking about names… so we were kind of scared to even make the plans…I would have wanted to do shopping for the new baby. That didn’t happen. I cried all the way to (the hospital).” [P3]

### Family support

Participants reflected on how the public health mandated separation from family resulted in missing out on much-needed support for them and the baby. One participant tried to use technology to bridge the gap, however she still felt disconnected, “we talked on the phone … [but] it cannot be compared with the physical presence of your family members when you’re eating together, laughing and sharing thoughts … It felt distant.” [P3]

Participants described breaking pandemic public health directives to access the emotional and physical family support that they needed: “at around two months I had to leave the city and go stay with my parents … it was definitely a feeling of unfairness, this isn’t fair. I wanted to have family, I wanted help.” [P9]

Another loss frequently expressed by the participants was the partner missing out on prenatal visits and imaging visits, “I think for him it was just kind of sad not to be super part of the experience it was just kind of sad for him not to get to see the ultrasounds and stuff.” [P11]

4) Challenges to Care

Participants described their access to care during pregnancy and postpartum care was compromised by the pandemic, especially breastfeeding support.

### Access

While some participants described enjoying aspects of virtual care, its inadequacies were a common theme. As this participant expressed, “Probably the most bothersome about this entire situation was I didn’t feel like I was getting the medical care that I needed or that the baby needed…you kind of feel like you’re being pushed aside a little bit.” [P9] As she explains, “I never had a six week postpartum appointment … I have not had a doctor look at me since I left the hospital … you’re on a virtual call and you can’t really explain things, you’re taking pictures that aren’t clear, it’s really, it’s not helpful.” [P9] Patients reported less access to IUD insertions and pelvic floor physiotherapy during pregnancy and postpartum as well.

Access to breast feeding support was negatively impacted due to the pandemic’s restrictions. As one participant reported, “We don’t have breastfeeding classes to go to and you don’t have in person… I was going to have a lactation consultant come to my home … I did it, virtually, once or twice and it was awkward and hard. I wanted someone right there with me. It’s just such a personal thing and they need to be right there to see so closely with the baby.” [P5]

Other participants had planned to attend a hospital breastfeeding class or in-home breast feeding support following delivery with either a lactation consultant or a midwife, however these too were cancelled due to pandemic restrictions. This left several of the first time parents with many questions regarding breastfeeding with very few opportunities for support.

### Quality

Some participants felt the quality of postpartum care was also compromised: “I did feel like my nursing care was – I don’t know if it was different because of the pandemic, or if it was just like workload, but I did feel like it wasn’t as ideal as I would have hoped, postpartum.” [P5]

Further, some participants felt that COVID-19 related topics overshadowed their health care visits, became the focus during their postpartum visits and thus the quality of care was impacted. One participant said, “you have all these questions, but it got so shadowed over by COVID.” [P6]

5) Strategies for Coping with Pandemic Stress

Participants developed different coping strategies to manage pandemic stress. Some described how they relied on or avoided information, while others participated in both adaptive and maladaptive behaviors.

### Approach to pandemic information

Some participants described themselves as information seekers. As one participant reported, “So paying more attention to the news, it was kind of like a distracter … I think that I’d rather know as much as I can.” [P9]

Conversely, most participants reported they avoided information overload to protect their mental health. As one participant described, information in the media was causing her heightened anxiety and she found she was better off not knowing, “I’ve been trying to avoid looking at the numbers as of late … Looking back I wish I hadn’t known. I think it would’ve been better for me to be kind of ignorant about it, because like it just gave me more anxiety than I was already feeling.” [P12]

### Adaptive behaviors

Participants described adaptive behaviors to help them cope, which included seeking support, exercise, self-care and finding a way to balance the risks and benefits of visiting with friends and family.

#### Seeking support

Participants described seeking support as helpful. One participant found that by speaking to her doctor she was reassured, “Definitely talking to my doctor about it … and then just sort of the reassurance that she was able to provide me.” [P2] Another participant found an online community that was supportive, “I’m in the one mom group … everybody’s going through stuff at the same time … it is definitely a place where I talk to other people just about how they’re dealing with things.” [P1]

#### Exercise

Many participants reported positive benefits on their mood and general well-being when they exercised. Some were able to find creative ways to stay active while maintaining social distancing protocols. Their strategies included online personal training, walking outside, using home gym equipment or meeting others at a park for an activity while maintaining social distancing protocols.

#### Self-care

It was common for participants to describe how important taking time to focus on themselves was for their mental health. As this participant explains, “I just turned mental health into my job … just really for once taking time for myself. A lot of self-talk too.” [P2] Another participant found listing what she is appreciative for as helpful, “every day I try to list what I’m grateful for. I get a lot of joy just out of the baby, like why have a baby if you’re not going to find her endlessly fascinating.” [P7]

#### Balancing exposure risks for the benefit of their mental health

Some participants decided to expand their bubble despite the risk of infection to themselves or their parents in order to surround themselves with the support they needed. One participant explained, “we decided it was more important to have people around, like COVID was a lower risk I guess than my mental health at that point.” [P10] One participant explained if she had not taken these actions, her mental health would have suffered, “We decided that the risk for my mental health was greater than the risk of them catching COVID from me, so I ended up going to stay with them [family] … If I didn’t have the ability to go and stay with them, I think that that would have been really detrimental.” [P9]

### Maladaptive behaviors

Some participants developed maladaptive behaviors during the pandemic, including decreased exercise, obsessively following strict hygienic practices and overeating.

#### Exercise

Some participants decreased the amount they exercised due to fear of leaving the home and risking exposure to the virus, “No, I wasn’t [exercising] because – oh my goodness, in my house? Where else? I didn’t want to go out.” [P3]

#### Hygiene practices

Participants described how their hygiene practices had changed due to the pandemic, with some becoming excessive. One participant shared her obsession with cleaning, “Wipe down the stroller and wash my hands, take off all his clothes. I don’t understand why I do that part, but I take off all his clothes, change him into a different set of clothes. … it makes me feel obsessed … Every minute you’re cleaning, because you just don’t know.” [P8]

#### Overeating

Stress eating was mentioned by some participants as a coping mechanism, “it’s one of the only things you can do at home, but it actually makes me feel worse … I’m just sitting at home and eating brownies, it definitely makes me feel worse.” [P1] See Table 2 for additional quotes supporting themes 1–5.

6) Advice

**Table 2:** Themes, subthemes and representative quotes derived from thematic analyses of participant interviews

Theme	Subtheme	Representative Quote
Childbearing-Related Challenges to Everyday Life	Employment	I think the biggest stress there was knowing I was going to be working from home and having a toddler and being super pregnant … was just a lot on my plate at the time, for sure. [P4]
	Access to Food	It’s just me doing everything right now. When I had the baby I had to come back and do my own groceries. I can’t call anyone. Because you don’t know who has what. My relatives, I couldn’t call. You just don’t know. [P8]
	Transportation	Going all the way down to Mount Sinai, you can’t take a bus on Jane Street, because the busses are still packed. There’s no distancing on that. I have to take the baby, I’d rather not. [P8]
Increased Worry, Uncertainty and Fear	Worry	I would think oh I’m going to take him to the library and read to him every day. And we’re going to go on walks and stuff and all that stuff was just out the window. I was very worried that he was going to have problems being social with strangers. […] But I’m sure there are lots of people who are not coping with this well at all. And it’s going to have a lot of long lasting effects for them and their children and their relationships. [P9]
	Uncertainty	So the unknown was really, really challenging. […] like no children have passed away from it but it’s really – I don’t know, it’s that unknown. It’s that fear of not understanding – like it hasn’t been around for so long that you understand and you can quantify what it is. [P6]
	Fear	I was kind of annoyed and a little bit angry and I was also concerned. Because my partner, my boyfriend, he was still working full time. And he didn’t have the option of taking the time off. So we were just - we didn’t know a lot about COVID and we were concerned that if he caught it, he would bring it back to me and then subsequently me and the baby. [P9]
Persistent and Pervasive Sense of Loss	Expectations vs Reality	I just felt robbed of the experience. [P4] I don’t know, you kind of have this idea of what your mat leave is going to be like and then it’s really not like that. [P1]
	Bonding With Baby	And it’s everybody’s first grandkid so they’re really definitely sad about missing out on this time… It just feels like they’re just babies for such a short time and … I don’t know, I feel like because we got to see what it was like before the lockdown, just seeing how much joy she brings everyone and how happy everybody is, and having them not be able to see her just really sucks. [P1]
	Family Support	I have my sister, I have my aunt, but because they were moving around, the thing is, they weren’t stopping their life, so I called them on the phone, but they wouldn’t come to me. You know, for us, the community and our culture is, we get together. We do family things. We meet every time. Every week we are together, we are eating, but now that was disconnected because they would not come, they are moving around, they have kids in daycare. So that was like gone because of the COVID. So we talked on the phone. That supposedly was support, but not as much as I wanted. [P3]
Challenges To Care	Access	I wasn’t able to go to pelvic floor physio which really was a bummer for a while because I think I have a lot of problems that would probably be helped by that and it just kind of sucked to – again, it’s just one of those things where it’s like everything you read, “Here’s what you should do to help with these things. You just can’t do those things.” So it was kind of upsetting for a while to feel like I know I needed help with this and needed some help with the healing side of things and I just couldn’t get it. [P1]
	Quality	I think there’s a real benefit of breastfeeding, like lactation consultants, or a midwife or someone helping you breastfeed in person. Because I did it, virtually, once or twice and it was awkward and hard. I wanted someone right there with me. It’s just such a personal thing and they need to be right there to see so closely with the baby. [P5]
Strategies for Coping with Pandemic Stress	Approach to Pandemic Information	*Seek out information*: What helped me is I do my own research. I read a lot. Sometimes it is good. Sometimes it is not good ... Yeah, because what I do is I panic, because, yeah, I seek information. I like reading from the reliable sources. That helps me a lot. [P3] *Avoid Information:* I’m an information seeker for sure but I found, with COVID, it was just almost too much information. [P5]
	Adaptive	I’m on the phone all the time, I guess that’s my coping mechanism. [P10]
	Maladaptive	I didn’t feel comfortable doing workouts on my own, because I wasn’t sure, you know, what was going to be safe, you know, monitoring my heart rate, things like that. [P2]

### Advice for other patients

The participants had several pieces of advice for other pregnant and postpartum individuals during a stressful period such as a pandemic. This advice included advocating for oneself and avoiding isolation.

#### Advocating for oneself

Participants stressed the importance of advocating for oneself in order to ensure adequate care, “I think that everyone’s doing a really good job with doing the best care they can, but I think it’s important to ask questions and seek out information about pregnancy or postpartum things, if you need it. I think that’s normal anyway, but I think, with the pandemic, it just makes it so much more necessary.” [P5]

#### Avoiding isolation

Participants focused on the importance of reducing isolation, “keep socializing as much as possible, bundle up and be outdoors as much as possible. Keep talking to people – friends, family, whoever. I mean those are strategies that worked for me, but I mean maternity leave can be a very isolating – I found it isolating without a pandemic at the same time. So even more so this time, I feel like we just have to rely on each other.” [P10]

Despite pandemic restrictions, participants emphasized the importance of reaching out for help, whether from family, friends, or health professionals: “having people that you can talk to, whether it’s a partner or family member, friend, a mental health professional … you don’t want to be holding those emotions inside of you, you want to find a release.” [P2]

### Recommendations for health care providers (HCPs)

Participants had recommendations for health care providers in order to help them improve care during a pandemic including: Providing clear and up to date information and increasing post-partum support.

#### Provide clear and up to date information

With the ever-changing recommendations and suggested precautions during a pandemic, participants felt there should be clear communication and up to date information coming from their HCPs. Specifically, they suggested clear messaging regarding hospital policies and what care would look like during their hospital stay, “it would be great if they had a better idea of what your stay is going to look like, so you’re a bit more mentally prepared for what’s to come.” [P10] One participant put it plainly, “there was a lot of confusion … try to be as clear to the patients as you can.” [P5] Moreover, participants wanted the health care team to have consistent messaging regarding in person versus virtual visit timing.

#### Increase postpartum support

Following discharge from hospital several participants voiced a need for more postpartum support. The suggestions as to what type of supports ranged from increased mental health support, increased breastfeeding support, more frequent maternal check ins to address any baby concerns as well as mom’s physical and psychological changes during the postpartum period. See Table 3 for additional quotes regarding advice.

**Table 3:** Participant’s advice for pregnant women and healthcare providers during COVID-19

		Representative Quote
**Advice for patients**	**Advocate for oneself**	You have to be your own best advocate because nobody is really advocating for you which sounds really depressing – because I don’t even mean it in a depressing way, I just don’t think anybody has time. You know, and I had, like – yeah, and just sort of be mindful of the fact that the system is overstretched so you have to really advocate for yourself and don’t be afraid to be annoying. [P7]
	**Avoid Isolation**	I think you need help at this time. So if you don’t have that, figuring out of you can have a friend or family member kind of come stay with you and be part of your bubble or … like if you feel you need help, find a way to get it right now. […] But I think if people are in that situation, you can’t just never see anyone and do this by yourself all the time. You need help somehow. [P1]
**Advice for HCPs**	**Provide Clear and Up to date Information**	So important because even if somebody else can’t deal with the unknowns for you, at least someone can give you the tools to sort of understand or like kind of develop. Because obviously mental health right now is first it’s identified, then it’s given, right; versus here is it, actually you don’t need it. Like it’s a different sort of thinking. So I think having the resources, “Saying yes, here’s your appointment. You know, the pandemic is happening, you have delivered, well your first appointment is for your baby, right after you get a wellness check as well of your mental health. And like how breastfeeding is going, you know, like how your own health is, like how are you recovering this and that” and then that would be good. [P6]
	**Increase Postpartum Support**	Even just not being able to go to physio felt really hard because it was like it was something that you need to do and I can’t do it right now. So just figuring out how – whether it’s online or with Zoom or whatever, that people can still get the support and the help that they need. [P1]

### Silver linings

Although the COVID-19 pandemic was a challenging time for our participants, several were able to find positives during the pandemic. Due to social distancing requirements, many participants had partners who were required to work at home. This enabled the partner to provide support and companionship as one participant explained, “His presence provided company and support … it was almost like a blessing in disguise.” [P2] Others appreciated the postpartum support from their husbands who were working from home, “having him being able to do more of the cooking and helping me get a nap every day.” [P1]

Other participants mentioned pregnancy and having a newborn provided a welcome distraction from the pandemic, “.. in some ways I think having a baby makes this whole situation easier. ..she really gives structure to my day. Like I have to get up in the morning, I have to do certain things for her. I just sort of focus on her and she’s changing all the time so it doesn’t feel like life has just stopped. So I think just finding the joy that you can in enjoying your baby and Face Timing a lot with family and friends. [P1]

## Discussion

The COVID-19 pandemic has created financial, logistical and emotional stresses for all people [35]. However, being pregnant or postpartum seemed to heighten these feelings as participants faced great uncertainty and fear for themselves and their babies during what is already typically a challenging life stage.

The COVID-19 pandemic impacted our participants’ emotional state in a variety of ways as they worried about their pregnancies and babies. During non-pandemic times one in seven pregnancies are marked by anxiety and depression during the perinatal period [36]. However, during past natural disasters and disease outbreaks, the prevalence of mental health disorders has been shown to increase [36–38]. One Canadian study of pregnant and postpartum women during the COVID-19 pandemic found higher levels of clinically significant depression and anxiety during the pandemic compared to pre-pandemic times [39]. Our participants experienced psychological stressors during their pregnancies and reported several emotional responses similar to that study. Of note the worries and fears of the participants from this study primarily focused on the consequences surrounding their baby’s health with less focus on themselves as well as the general uncertainty of the pandemic as a whole. HCPs should recognize this concern and address it early on in order to help support the pregnant individual. It is important to note that our study recruited participants as the province was slowly easing its restrictions and returning to some sense of normalcy from August to November 2020. Prior studies have shown that longer periods of quarantine are associated with more severe psychological distress [38].

As the participants were required to socially distance from their friends and most of their families, they experienced a profound sense of loss. Their hopes and expectations of what their pregnancy and postpartum period could have been were dashed, their ability to bond with their babies was impacted and they expressed the tremendous loss of not being able to access important support from family. Ceremonies and rituals typically held during pregnancy and the postpartum period including showers and religious ceremonies were cancelled or scaled back. These social gatherings have been shown to strengthen the family’s connection and help the postpartum person’s mental health [40].

Pregnant and postpartum individuals are more likely to feel the effects of isolation [41] compared to non-pregnant individuals and isolation may lead to distress over time [42]. The importance of social support is further explained when considering the four types of social support: esteem, instrumental, informational and social companionship [43, 44]. Two types of support stand out as particularly essential to a new parent and are lacking during COVID-19 due to imposed distancing measures. These include esteem support that raises parental self-esteem and confidence necessary to undertake the role of parent as well as instrumental support that describes resources including help with childcare. Our participants described an intense impact from this lack of support from family and friends due to social distancing requirements. Pregnant individuals tend to experience higher levels of anxiety and depression when socially isolated or if they feel they have low social support [41, 45]. Despite lacking the different form of in person support, it is imperative during a pandemic lockdown that pregnant and postpartum women find ways to feel emotionally supported. This can be achieved by using virtual platforms in order to access and interact with family, friends and health care providers. As suggested in a prior study [5], another option shown to have a preventative effect for post-partum depression is to recruit individuals with prior experience with psychological distress to provide peer support to those that are vulnerable during pandemic times [46].

This study has focused primarily on the pregnant and postpartum individual’s loss; however, another important consideration is the effect of restrictions which limit the partner’s active participation in the prenatal and postpartum experience. Limiting the partner’s involvement has been shown to impede the partner’s ability to engage with their newborn which may impact early father-baby attachment and the overall family dynamic [47]. Considerations should be made to ensure the family unit is kept together as much as possible during the pregnancy and postpartum period. If physical distancing is enforced, then using creative ways such as handheld devices to video call during medical appointments so that the partner can be present from afar should be considered.

Not only did pregnant individuals lose critical support people, but the participants in this study described being negatively impacted by what they felt was both decreased access to and quality of care. Like many other institutions our center quickly implemented a hybrid care model with staggered virtual and in-person visits throughout the span of a pregnancy in order to adhere to social distancing protocols. In the early stages there was little evidence as to the effectiveness of prenatal virtual care. One study has described positive factors associated virtual prenatal visits including convenience and improved access. However, this same study also highlights the negative factors from both the patient and providers’ perspectives including access inequities, poor virtual visit quality and safety without home devices including blood pressure cuffs and poor patient-provider continuity [48].

The implementation of virtual visits can also disrupt a pregnant individual’s expectations of her prenatal care. This too has been shown to cause stress to pregnant individuals. A study exploring the effects of alterations to expected prenatal care during the COVID-19 pandemic coined the term “preparedness stress” and reported that one in three pregnant women developed increased stress with changes in their care [49, 50].

In order to offset the uneasiness of virtual visits as well as help pregnant people feel more prepared for the transition to parenthood health care providers may want to reconsider how and when they implement virtual care schedules to ensure that their patients receive both the medical care and psychosocial support they require. Furthermore, hospitals should consider using technology when possible to lessen the impact of social distancing. Medical teams offering prenatal care and postpartum units could consider using online resources, virtual hospital tours and, online perinatal education classes to help educate and allow the pregnant individual to feel more prepared [51].

Our participants reported both information seeking and information avoidance behavior. Information seeking to fill knowledge gaps is a behavior that has been described in pregnant people during the COVID-19 - pandemic. Specifically, one study found pregnant people desired official information from the hospital regarding virus transmission risk, precautions being taken to reduce risk as well as clarification on any changes to delivery protocols [50]. Moreover, for these individuals, if they are not able to fill these gaps, feelings of uncertainty and perceived risk of getting COVID-19 can amplify [2]. Whether a pregnant individual choses to seek out or avoid information in order to cope, it is nearly impossible to avoid any incoming information. Health care providers should focus on effective means of communicating with their patients. Prior to disseminating any information, HCPs must determine the needs of their patients with respect to type of information they would like to receive, their literacy levels and type of communication medium the pregnant individuals are comfortable with [27]. It remains an ongoing challenge for HCPs to provide accurate and up to date information in a clear way as recommendations continue to evolve during a pandemic while researchers continue to learn more about the virus and its impact. HCPs should do their best to keep abreast of changes that apply to their patients and their care with the understanding that what seems self-evident today was a novel and controversial concept yesterday.

Decreased exercise and overeating, largely related to being confined to the home, were some maladaptive behaviors participants described. Other studies have reported similar behavior by pregnant individual during the pandemic [40, 52]. Although pandemic restrictions such as recreational facility closures make it more challenging to reach the recommended 150 minutes of moderate-intensity physical activity per week [8] in pregnancy, physical activity should continue to be encouraged by health care providers. Health care providers may consider recommending resources such as videos and websites that individuals may use at home or outdoor activities where social distancing is possible. Canadian studies suggest that pregnant and postpartum women who were able to participate in physical activity during COVID-19 may have improved mental health compared to those who did not maintain activity [39]. (Kolker S, Biringer A, Bytautas J, Kukan S, Carroll J: Identifying the psychological impact and behavioural changes of pregnant women during COVID-19, in progress).

Many of the participants demonstrated adaptive behaviors as they sought out ways to cope. Of note was seeking out support, whether it be with their HCP, family and friends or online network, exercising and engaging in self-care. These same protective coping behaviors amongst pregnant individuals have been reported in past studies [28, 53, 54]. The importance of social support is reemphasized here as being an essential component to managing stress during the perinatal period.

Pregnant and postpartum individuals in this study faced significant emotional challenges, a profound sense of loss, lack of support and challenges to care, similar to those described in other studies [28, 55, 56]. Pregnant individuals require additional support, guidance and access to care during pandemic times in order to diminish their psychological distress. The challenges of providing care during pandemic times will continue to be an issue due to social distancing restrictions; however, every member of the health care team should take advantage of every contact point, either in person or virtually to check in with the patient regarding their mental health. It is also important to recognize how crucial social support is during the postpartum period. Postnatal depression has been shown to be inversely associated with social support, more specifically individuals with more depressive symptoms report lower social support [26, 57]. One study looking at the longitudinal effects of social support on perinatal and postpartum depression suggests focusing on perinatal depression treatment approaches from late pregnancy to the first 6 months postpartum [26]. This further underscores how HCPs should recognize the importance of support at every stage of the pregnant and postpartum period.

### Limitations

The main limitation of our study is that our participants were well educated, of high socioeconomic status, employed and partnered. Thus it is difficult to generalize these findings to other populations. However, despite their advantages, these people still struggled with the effects of the pandemic in multiple ways during their pregnancies and postpartum period. Our study is unique in that it directly captures their lived experience and experiences of individuals who were pregnant and postpartum during the pandemic. To our knowledge no study during COVID-19 has added to the literature by asking patients directly to provide advice to others in their same situation as well as their health care team.

## Conclusion

This study enhances our understanding of pregnant and postpartum individuals’ lived experiences during the COVID-19 pandemic. Our findings reveal heighted emotional responses, a profound sense of loss centered around lack of support and challenges to access and quality of care. As a result the participants developed coping mechanisms to navigate these pandemic stressors. By specifically inquiring about how to improve the pregnancy and postpartum pandemic experience, this study provides a deeper understanding of positive changes both patients and HCPs can make during the antenatal and postpartum period. Applying this advice may provide important psychosocial benefits to both the pregnant individual, the baby and the family unit as they navigate through these challenging times.

## Data Availability

The datasets obtained during the current study is not publicly available due to confidentiality but may be available from the corresponding author on reasonable request.
